# DEPTOR maintains plasma cell differentiation and favorably affects prognosis in multiple myeloma

**DOI:** 10.1186/s13045-017-0461-8

**Published:** 2017-04-18

**Authors:** Dalia Quwaider, Luis A. Corchete, Irena Misiewicz-Krzeminska, María E. Sarasquete, José J. Pérez, Patryk Krzeminski, Noemí Puig, María Victoria Mateos, Ramón García-Sanz, Ana B. Herrero, Norma C. Gutiérrez

**Affiliations:** 1 Cancer Research Center-IBMCC (USAL-CSIC), Salamanca, Spain; 2 Department of Hematology, University Hospital of Salamanca, Paseo San Vicente, 58-182, Salamanca, 37007 Spain; 3 Institute of Biomedical Research of Salamanca (IBSAL), Salamanca, Spain; 4 National Medicines Institute, Warsaw, Poland

**Keywords:** DEPTOR, Multiple myeloma, Plasma cell development, B lymphocyte differentiation, miRNAs

## Abstract

**Background:**

The B cell maturation process involves multiple steps, which are controlled by relevant pathways and transcription factors. The understanding of the final stages of plasma cell (PC) differentiation could provide new insights for therapeutic strategies in multiple myeloma (MM). Here, we explore the role of DEPTOR, an mTOR inhibitor, in the terminal differentiation of myeloma cells, and its potential impact on patient survival.

**Methods:**

The expression level of DEPTOR in MM cell lines and B cell populations was measured by real-time RT-PCR, and/or Western blot analysis. DEPTOR protein level in MM patients was quantified by capillary electrophoresis immunoassay. RNA interference was used to downregulate DEPTOR in MM cell lines.

**Results:**

DEPTOR knockdown in H929 and MM1S cell lines induced dedifferentiation of myeloma cells, as demonstrated by the upregulation of *PAX5* and *BCL6*, the downregulation of *IRF4*, and a clear reduction in cell size and endoplasmic reticulum mass. This effect seemed to be independent of mTOR signaling, since mTOR substrates were not affected by DEPTOR knockdown. Additionally, the potential for DEPTOR to be deregulated in MM by particular miRNAs was investigated. The ectopic expression of miR-135b and miR-642a in myeloma cell lines substantially diminished DEPTOR protein levels, and caused dedifferentiation of myeloma cells. Interestingly, the level of expression of DEPTOR protein in myeloma patients was highly variable, the highest levels being associated with longer progression-free survival.

**Conclusions:**

Our results demonstrate for the first time that DEPTOR expression is required to maintain myeloma cell differentiation and that high level of its expression are associated with better outcome.

Primary samples used in this study correspond to patients entered into GEM2010 trial (registered at www.clinicaltrials.gov as #NCT01237249, 4 November 2010).

**Electronic supplementary material:**

The online version of this article (doi:10.1186/s13045-017-0461-8) contains supplementary material, which is available to authorized users.

## Background

Multiple myeloma (MM) is a clonal disorder of B cells (BCs) in the final stage of differentiation that accounts for approximately 10% of all hematological cancers [1]. MM is characterized by clonal accumulation of malignant plasma cells (PCs) in the bone marrow, which secrete a monoclonal immunoglobulin. Although several therapeutic agents are available, MM remains incurable. Knowledge of all the factors involved in PC differentiation could provide new insights of relevance to therapeutic strategies for MM. In fact, in some hematological neoplasms, the malignant transformation of BC has been associated with the disruption of the B cell differentiation process, such as mutations of certain key BC maturation factors [2–4]. The transition from B lymphoid precursors to antibody-secreting PCs involves several molecular and cellular modifications including transcriptional changes, expansion of the cytoplasm and the secretory organelles to accommodate high-rate synthesis of immunoglobulins, unfolded protein response (UPR) activation, and changes in cell surface antigen expression [5–8]. It has been demonstrated that the transcriptomes of BC and PC are maintained by two groups of transcriptional factors: those that promote the B cell program, such as PAX5, BCL6, and BACH2, and those that favor and facilitate PC differentiation, notably IRF4, BLIMP1, and XBP1 [9]. Interestingly, many of these transcription factors repress others required for the alternative developmental state, thereby establishing mutually exclusive gene expression programs [9–12]. Besides transcriptional factors, other types of proteins and biochemical pathways could be involved in the transformation of BC into mature PC.

Using microarray expression data, we found that the mRNA-encoding DEPTOR, an inhibitor of mTORC1 and mTORC2 kinases activities [13], was overexpressed in normal PCs (NPCs) and myeloma cells compared with normal B lymphocytes (NBLs) [14], which raised the possibility that this protein contributes to PC differentiation. The complete role of DEPTOR within the cells has not yet been fully elucidated, although the involvement of DEPTOR in several biological processes, such as cell growth, apoptosis, and autophagy, has been reported [15]. A potential role of DEPTOR as a tumor suppressor or as an oncogene, depending on cell context, has also been described. It is considered a tumor suppressor, functioning by the inhibition of mTOR, whose activity is frequently hyperactivated in many human tumors. Indeed, DEPTOR has been found to be downregulated in many types of human cancers. However, it is also overexpressed in many other tumor types, including chronic myeloid leukemia, and MM [13, 16]. The overexpression of DEPTOR in MM has been associated with translocations involving MAF transcription factors and *CCND1* and *CCND3* genes [13]. Furthermore, DEPTOR seems to be overexpressed in MM with copy number gains of 8q24 where DEPTOR is located [17].

Here, we report for the first time that DEPTOR maintains the terminal differentiation of MM cells. Knockdown of DEPTOR reverts the transcriptional program of the PC to that characteristic of a BC. In addition, we found that microRNA deregulation in MM, specifically miR642a and miR135b downregulation, may also underpin the overexpression of DEPTOR.

## Methods

### Cell lines and primary samples

The human multiple myeloma cell lines (MMCL), NCI-H929, MM1S, and U266 were acquired from the ATCC (American Type Culture Collection), and the JJN3, RPMI-8226, OPM-2, KMS12BM, KMS12PE, and HEK923 lines were obtained from the Deutsche Sammlung von Mikroorganismen and Zellkulturen (DSMZ). Cell line identity was confirmed periodically by STR analysis with the PowerPlex 16 HS system kit (www.promega.com) and online STR matching analysis (www.dsmz.de/fp/cgi-bin/str.html). Cell lines were cultured in RPMI 1640 medium supplemented with 10% fetal bovine serum and antibiotics (Gibco Life Technologies, Grand Island, NY, USA). Bone marrow (BM) samples from ten healthy donors were sorted by a FACSAria equipment into four BC populations: immature B cells (CD34−, CD19 +, CD10+, CD38++), naive B cells (CD19+, CD27−, CD10−), memory B cells (CD19+, CD138−, CD27+, CD38+), and plasma cells (CD38+++, CD138+, CD45low). Monoclonal antibodies were purchased as follows: anti-CD45-FITC (clone D3/9) and anti-CD19-PECy7 (clone A3-B1) from Immunostep (Salamanca, Spain); anti-CD38-PerCP-Cy™5.5 (clone HIT2), anti-CD34-APC (clone 8G12), and anti-CD27-BV421 (clone M-T271) from BD Biosciences (San Jose, CA, USA); anti- CD138-Pacific OrangeTM (clone B-A38) from Exbio Praha (Vestec, Czech Republic); and anti-CD10-PE (clone ALB1) from Beckman Coulter (Pasadena, CA, USA). CD138+ plasma cells were isolated from BM samples of 24 patients with newly diagnosed MM included in the GEM2010 Spanish trial (bortezomib, melphalan, and prednisone plus lenalidomide and dexamethasone), using an autoMACS separation system (Miltenyi-Biotec, Auburn, CA, USA).

### RNA extraction and quantitative real-time PCR analysis

RNA was extracted from the cell lines using an RNeasy mini kit (Qiagen, Valencia, USA) according to the standard protocol. RNA integrity was assessed using an Agilent 2100 Bioanalyzer (Agilent Technologies, Santa Clara, CA, USA). Total RNA (1 μg) was reverse-transcribed to complementary DNA (cDNA) using High-Capacity cDNA Reverse Transcription Kits (Applied Biosystems, Foster City, CA, USA). Expression of target genes was assessed using TaqMan qRT-PCR assays (Applied Biosystems). Relative gene expression was calculated by the 2^−ΔCt^ method using GAPDH as the endogenous control for normalization.

To detect mature miR135b and miR642a expression levels, TaqMan quantitative real-time polymerase chain reaction (qRT-PCR) micro RNA (miRNA) assay (Applied Biosystems) was performed. The relative levels of expression of mature miR135b and miR642a normalized with respect to the RNU43 endogenous control were determined by the 2^−ΔCt^ method. Each measurement was performed in triplicate.

### Transfections

Cell lines were transfected using the nucleofector II system (Lonza, Allendale, NJ, USA) with the following programs: C-16 for H929 and JJN3, G-16 for MM1S, and X-005 for U266. Cells were transfected with on-TARGET plus™ control pool or on-TARGET plus SMART pool Human DEPTOR (Dharmacon, Lafayette, CO, USA); pre-miR™ miRNA precursors pre-miR-135b, pre-miR-642a, and pre-miR™ miRNA negative non-targeting control#1 (Ambion, Austin, TX, USA); and microRNA inhibitors, hsa-miR-135b-5p miRCURY LNA™ microRNA inhibitor, hsa-miR-642a-5p miRCURY LNA™ microRNA inhibitor, and miRCURY LNA™ microRNA inhibitor negative control A (Exiqon, Woburn, MA, USA). Small interfering RNA (siRNA) and miRNA concentration of 25 nM was used in all the experiments.

### Cell cycle analysis

Cells were washed in PBS and fixed in 70% ethanol for later use. Cells were rehydrated with PBS, resuspended in 500 μl of PI/RNase staining solution (Immunostep), and incubated for 20 min at RT in the dark. Samples were analyzed using a FACSCalibur flow cytometer.

### Apoptosis and cell proliferation assays

Apoptosis was measured using an annexin V-fluorescein isothiocyanate/propidium iodide (PI) double staining (Immunostep) according to the manufacturer’s procedure. Cell viability was evaluated with the CellTiter-Glo® luminescent cell viability assay based on the amount of ATP present (Promega), in accordance with the manufacturer’s protocol.

### Immunophenotyping

MM cell lines were immunophenotyped on a FACSCanto II cytometer (Beckton Dickinson Biosciences) using the following monoclonal antibodies: CD138-OC515 (Cytognos S.L., Salamanca, Spain), CD38-APC-H7 (BD Biosciences), and sIgk-PB (Vestec, Czech Republic). Data analysis was performed using the Infinicyt software (Cytognos S.L.). A minimum of 10^5^ events were stored. Median fluorescence intensity of each marker was analyzed.

### Luciferase reporter assay

The double-stranded oligonucleotides corresponding to the wild-type or mutant miR135b and miR642a binding sites in the 3′-untranslated region (3′UTR) of *DEPTOR* were synthesized (Additional file 1: Table S1) (Sigma-Aldrich, St Louis, MO, USA) and ligated between the *Pme*I and *Xba*l restriction sites of the pmirGLO vector (Promega, Madison, WI, USA). Oligonucleotide sequences are detailed in Additional file 1: Table S1. Luciferase assays in HEK293 cells were performed as previously described [18].

### Western blot

Protein extraction and Western blot analysis were carried out as previously detailed [18]. The primary antibodies used for immunoblotting were anti-DEPTOR, anti-AKT, phospho-AKT (ser473), anti-p70 S6 kinase, anti-phospho-p70 S6 (Thr389), anti-4E-BP1, anti-phospho-4E-BP1 (Thr37/46), anti-S6 ribosomal protein, anti-phospho-S6 ribosomal protein (Ser235/236) (Cell Signaling Technology, Beverly, MA, USA), anti-IRF4, anti-Ig kappa light chain, and anti-Ig lambda light chain (Santa Cruz Biotech, Delaware, CA, USA). Anti-β-actin (Sigma-Aldrich) was used as an internal control for protein loading. The membranes were then washed and incubated with the secondary horseradish per-oxidase-linked anti-mouse IgG or anti-rabbit IgG antibodies (PierceNet) (1:10000), anti-goat IgG (Santa Cruz Biotech) (1:10000). Chemiluminescence was detected using the Amersham ECL Plus™ Western Blotting Detection Reagent (GE Healthcare).

### Capillary electrophoresis immunoassay

Capillary electrophoresis immunoassay was performed using the WES™ machine (ProteinSimple Santa Clara, CA, USA) according to the manufacturer’s protocol. In brief, 4 μl of samples at a concentration of 0.1 mg/ml (or lower when it was not possible to achieve 0.1 mg/ml) were combined with a master mix (ProteinSimple) to a final concentration of 1× sample buffer, 1× fluorescent molecular weight markers, and 40 mM dithiothreitol (DTT), and then heated at 95 °C for 5 min. The samples, blocking reagent, wash buffer, primary antibodies (anti-DEPTOR and anti-GAPDH at 1:100 concentration), secondary antibodies, and chemiluminescent substrate were dispensed into designated wells in the microplate provided by the manufacturer. After plate loading, the fully automated separation electrophoresis and immunodetection steps were carried out in the capillary system. Data were analyzed with the inbuilt Compass software (ProteinSimple). The signal from DEPTOR was normalized with respect to the signal from GAPDH, making sure that the signals of both proteins were within the linearity range.

### Immunofluorescence staining

Cells were collected 48 or 72 h post-transfection, washed with PBS, and stained for 30 min with 1 μM ER-Tracker™ Red (Invitrogen). Cells were washed again with PBS, fixed with 4% formaldehyde for 5 min at room temperature, placed on glass slides coated with poly-l-lysine, and stained for 1 min with DAPI II. The slides were then mounted using VECTASHIELD Mounting Medium (Vector Laboratories, Burlingame, CA, USA). Images were collected under a Zeiss confocal microscope equipped with 636/1.4 Oil Plan-APOCRHOMAT DIC.

### Statistical analysis

The two-tailed Student *t* test or the two-tailed Welch *t* test was used to analyze group differences in experiments when data showed equal or unequal variances, respectively. Data are reported as mean values ± standard deviation (SD) of at least three determinations.

Progression-free survival (PFS) distribution curves were plotted using the Kaplan–Meier method; the log-rank test was used to estimate the statistical significance of differences between the curves. The Cutoff Finder web application (http://molpath.charite.de/cutoff) was used to determine the optimal cutoff, defined as that yielding the most significant split discriminating shorter and longer survival, and identified by testing all possible cutoffs using the log-rank test [19]. Values of *p* < 0.05 were considered statistically significant. All statistical analyses were conducted using the SPSS 21.0 program (IBM Corp. Released 2012. IBM SPSS Statistics for Windows, Version 21.0. Armonk, NY: IBM Corp).

## Results

### DEPTOR is overexpressed in plasma cells compared with B lymphocytes

**Fig. 1:**
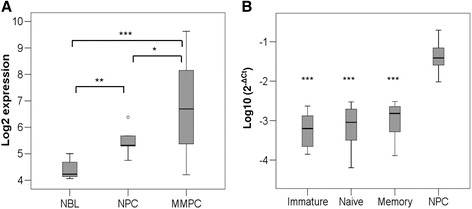
DEPTOR expression. **a** *DEPTOR* expression in normal B lymphocytes (NBL), normal PC (NPC), and myeloma cells obtained from gene expression arrays (GSE6691 at GEO repository). **b** *DEPTOR* expression detected by qRT-PCR in immature B cells, naive B cells, memory B cells, and NPC. (*p ˂ 0.05, **p ˂ 0.01, ***p ˂ 0.001)

In order to confirm the previously observed overexpression of *DEPTOR* by microarray analysis in NPC and myeloma cells relative to NBL (GSE6691 at GEO repository) [14] (Fig. 1a), we quantified *DEPTOR* mRNA levels by qRT-PCR in four BC populations. To this end, immature, naïve, memory B cells and PCs were sorted from BM samples obtained from healthy donors. *DEPTOR* expression was found to be significantly higher in PCs than in all previous stages of differentiation (Fig. 1b), which suggested that this protein could be involved in PC maturation.

### DEPTOR knockdown induces dedifferentiation of myeloma cells

**Fig. 2:**
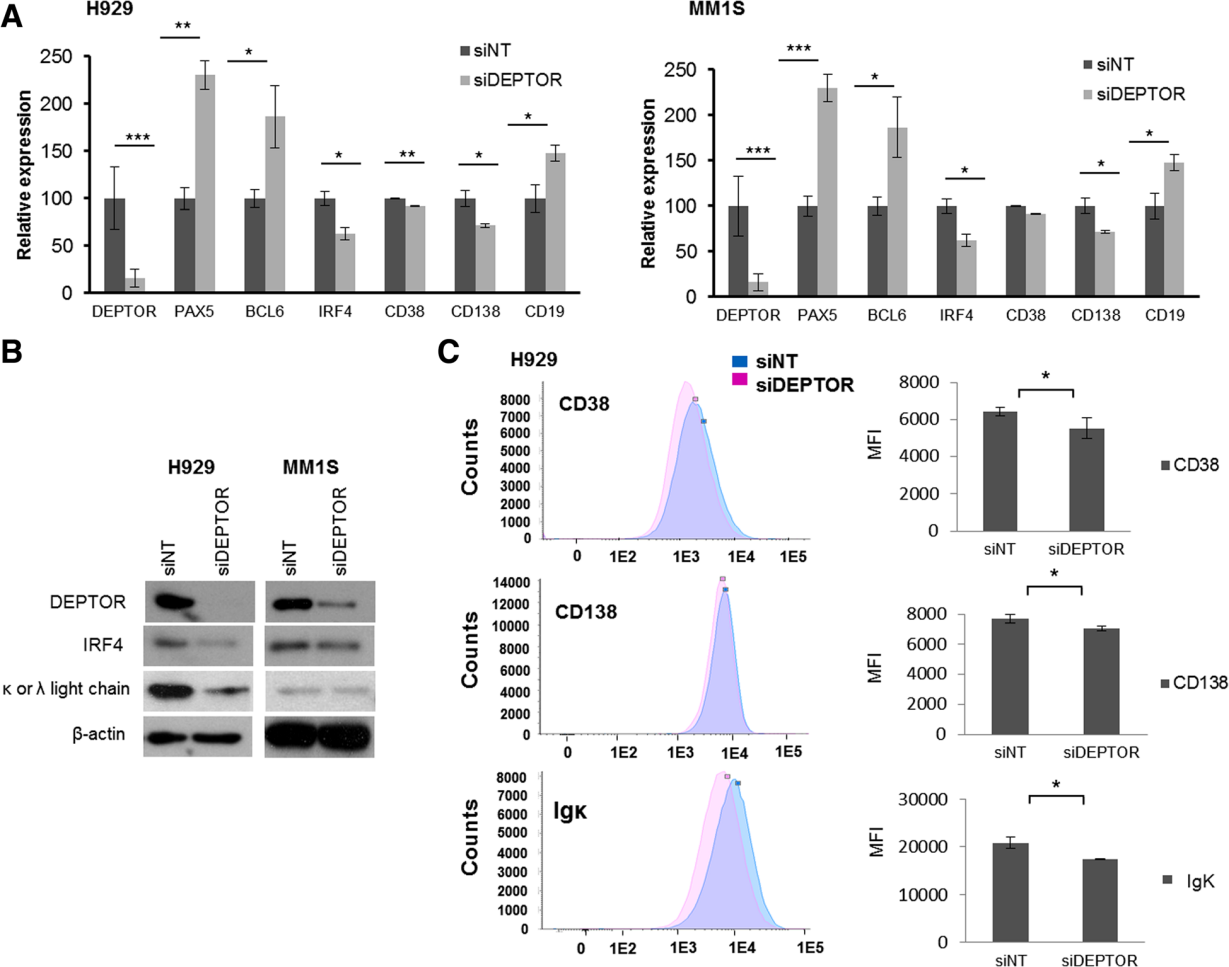
DEPTOR knockdown modulates transcription factors and markers related to B cell maturation. **a** mRNA levels of the indicated genes in H929 and MM1S determined by qRT-PCR 48 h after DEPTOR knockdown. **b** Western blot of DEPTOR, IRF4, and kappa and lambda light chain in H929 and MM1S. **c** Flow cytometry analysis of CD38, CD138, and cytoplasmic kappa light chain in H929 after DEPTOR knockdown (siDEPTOR) compared with non-targeting control (siNT); *left panel*: representative histograms; *right panel*: mean fluorescence intensity values (MFI). All results are presented as the means ± SD of three different experiments. (*p ˂ 0.05, **p ˂ 0.01, ***p ˂ 0.001)

**Fig. 3:**
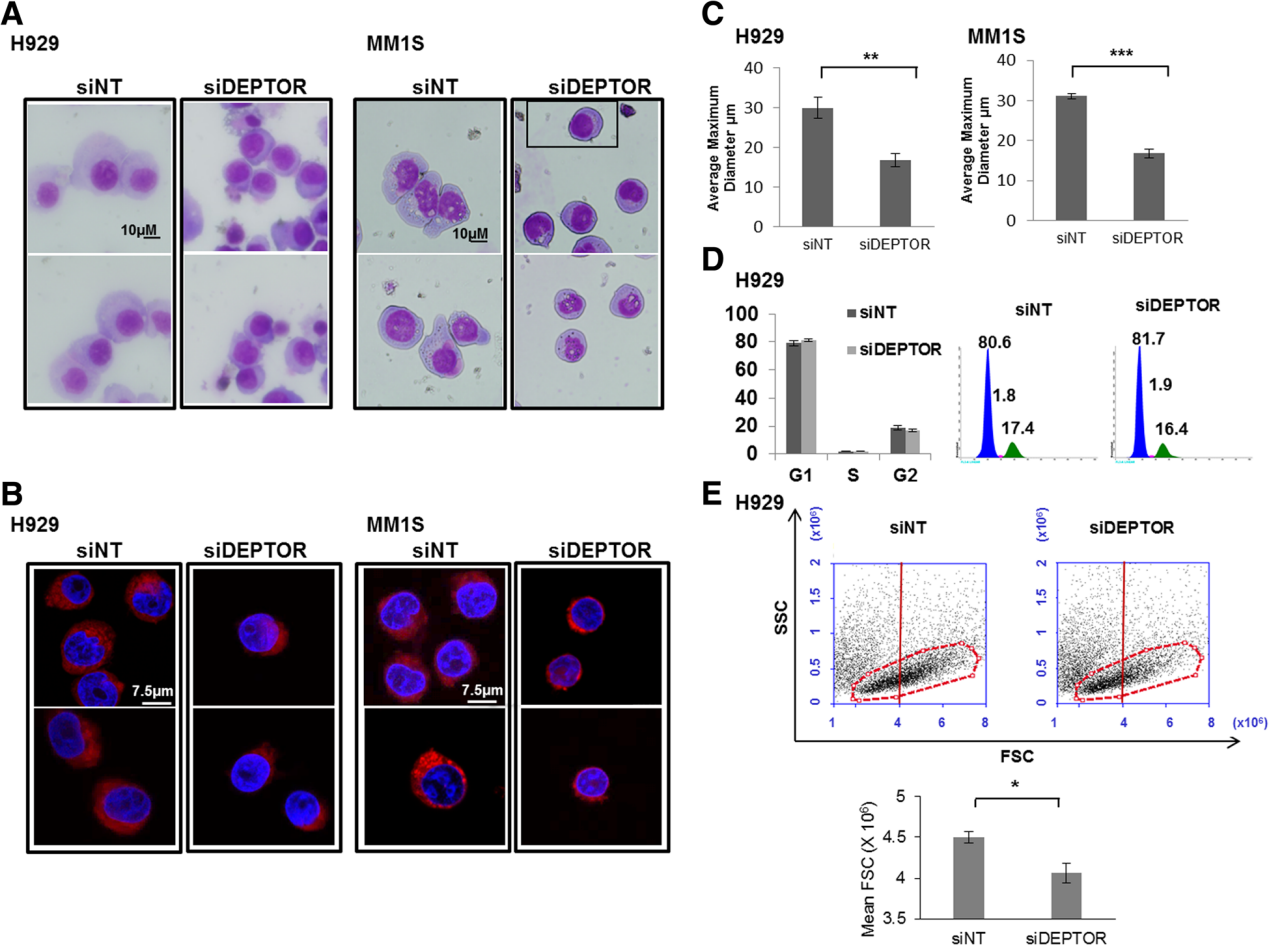
DEPTOR knockdown induces dedifferentiation of myeloma cells. **a** Giemsa stain of H929 and MM1S after DEPTOR knockdown. **b** Immunofluorescence of H929 and MM1S cells stained with ER-Tracker™ Red dye. **c** Average maximum diameter of H929 and MM1S transfected with non-targeting (siNT) and DEPTOR siRNA (siDEPTOR) measured from two independent experiments. At least 100 cells per experiment were counted. **d** Cell cycle analysis 48 h after DEPTOR knockdown. The percentages of cells in different phases of the cell cycle are indicated. **e** Cell size of H929 48 h after DEPTOR knockdown. Representative dot plots showing forward-scatter (FSC) versus side-scatter (SSC). All results are presented as the means ± SD of three different experiments. (*p ˂ 0.05, **p ˂ 0.01, ***p ˂ 0.001)

To gain further insight into the potential role of DEPTOR in PC differentiation, we knocked down its expression in MMCLs by siRNA for 48 h and then assessed the expression of key genes involved in B cell maturation by qRT-PCR. Cell growth and apoptosis experiments revealed that DEPTOR knockdown did not alter cell viability (Additional file 1: Figure S1 a and b). However, we found that *PAX5* and *BCL6*, which both encode B cell lineage-specific activator proteins and which are present only at early stages of B cell differentiation [9], were increased in DEPTOR-silenced cells (Fig. 2a). Conversely*,* IRF4, an essential transcription factor for PC differentiation [20], was downregulated after DEPTOR knockdown. Reduced IRF4 levels were also confirmed by Western blot, mainly in H929 cells (Fig. 2b). Additionally, immunophenotypic markers related to B cell differentiation, such as CD19, CD38, CD138, and k light chain were also assessed. Thus, expression levels of CD38, CD138, and k light chain were found to be lower, while CD19 expression was higher after DEPTOR silencing (Fig. 2a–c). Next, we analyzed the effect of DEPTOR downregulation on myeloma cell morphology. Clear reductions in cell size and endoplasmic reticulum (ER) mass were found in both H929 and MM1S as a consequence of DEPTOR downregulation (Fig. 3a–c). To exclude the possibility that cell cycle profiles were responsible for the differences in size of myeloma cells, we determined the percentage of cells in G1, S, and G2/M by flow cytometry. No differences in cell cycle profiles were observed between control and DEPTOR-silenced cells (Fig. 3d). Next, we also determined whether the observed reduction in cell size in DEPTOR-silenced cells could also be detected by flow cytometry. A clear reduction in mean FSC value was observed in DEPTOR-silenced cells compared with control cells (Fig. 3e). Taken together, these results indicate that DEPTOR downregulation in MM cells induces a reversal of PCs to previous stages of PC differentiation.

### Dedifferentiation of myeloma cells induced by DEPTOR silencing is independent of mTOR signaling

**Fig. 4:**
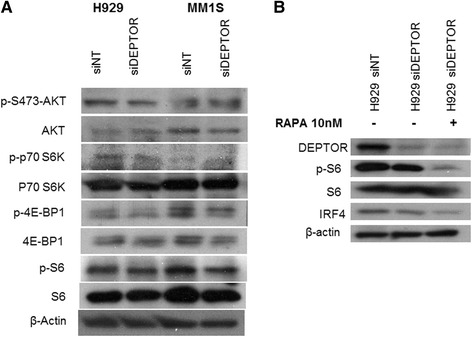
Dedifferentiation of myeloma cells after DEPTOR silencing is independent of mTOR signaling. **a** Levels of mTORC1 and mTORC2 substrates after DEPTOR knockdown in H929 and MM1S. All experiments were carried out 48 h post-transfection. **b** Western blot of H929 transfected with siNT or siDEPTOR and cultured with control medium and rapamycin at 10 nM

As DEPTOR has been described previously as an mTORC1/mTORC2 inhibitor [13], we were interested to determine whether PC dedifferentiation observed after DEPTOR silencing was induced through the mTOR signaling pathway. For this purpose, mTORC1 and mTORC2 activity was monitored through the phosphorylation state of their substrates using the same conditions of DEPTOR knockdown that induced PC dedifferentiation. We found that the levels of mTOR substrates were not changed by DEPTOR knockdown under the conditions assayed (Fig. 4a). These results implied that all the molecular and morphological changes obtained after DEPTOR knockdown were independent of the mTOR pathway. To confirm these findings, we added rapamycin, a well-known mTORC1 inhibitor, in the DEPTOR knockdown experiments. As shown in Fig. 4b, levels of p-S6 were again found to be similar in control and in DEPTOR knockdown cells, but lower in cells exposed to rapamycin, showing that the downregulation of IRF4 triggered by DEPTOR silencing was not reverted by the addition of rapamycin. Therefore, the PC state is maintained by DEPTOR independent of its role as an mTORC inhibitor.

### DEPTOR is a direct target of miR135b and miR642a

**Fig. 5:**
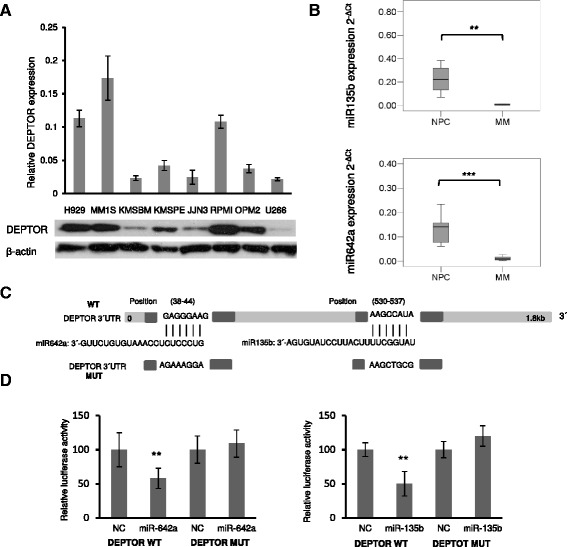
DEPTOR expression is controlled by miR135b and miR642a in MM. **a** *DEPTOR* mRNA levels measured by qRT-PCR (*upper panel*). Levels of DEPTOR in MM cell lines detected by Western blot (*lower panel*). **b** miR135b and miR642a expression in NPC and MM patients measured by qRT-PCR. microRNA expression was normalized to RNU43 (2^−Δct^). Two-tailed Welch’s *t* test *p* values for miR135b and miR642a were calculated (0.0081 and 0.0001, respectively). **c** Schematic diagram of the miR642a and miR135b predicted site on the *DEPTOR* 3′UTR, dark squares on *DEPTOR* 3′UTR WT, and *DEPTOR* 3′UTR MUT represents fragments cloned in pmiRGlo plasmid. **d** Luciferase activity in HEK293 cells cotransfected with pre-miR-NC or pre-miR135b/ miR642a and plasmid pmiR-Glo with the putative miR135b/miR642a binding site of *DEPTOR* cloned downstream of the luciferase reporter gene. Luciferase activity was normalized using Renilla. All results are presented as the means ± SD of three different experiments. (*p ˂ 0.05, **p ˂ 0.01, ***p ˂ 0.001)

It has previously been shown that DEPTOR is overexpressed in the subset of MM carrying *MAF/MAFB* and *CCND1/CCND3* translocations. The influence of MAF/MAFB on DEPTOR levels was confirmed by both ectopic expression of MAFB and MAF silencing, which induced DEPTOR upregulation and downregulation, respectively [13]. We evaluated DEPTOR expression in a panel of MM cell lines with different chromosomal translocations (Fig. 5a). DEPTOR mRNA and protein levels were detected in almost all cell lines analyzed, especially in RPMI-8226 and MM1S, which overexpress MAF [21], but also in H929 and OPM2 carrying the (4;14) translocation. Surprisingly, some MM cell lines exhibited low levels of DEPTOR, like JJN3, even though it carries a *MAF* translocation, and U266, which overexpress cyclin D1 [22–26]. These results suggest that, although DEPTOR levels may be influenced by MAF/MAFB or cyclin D1/3 expression, additional mechanisms may affect its expression. To address the possibility that post-transcriptional regulation by microRNAs is involved in DEPTOR expression, we looked for miRNA-*DEPTOR* interactions in five databases (miRMap, PITA, RNA22, RNAhybrid, and Targets scan), using a value of *p* = 0.05 for miRNAs whose predicted binding site is the 3′UTR of DEPTOR (Additional file 1: Figure S2). A total of 47 common miRNAs were predicted to target *DEPTOR* from the five combined datasets. Of these, miR135b and miR642a had been previously reported to be downregulated in MM with different cytogenetic abnormalities [27]. Underexpression of both miRNAs in different MM patients compared with NPCs was confirmed by qRT-PCR (Fig. 5b). According to the prediction algorithms, miR642a and miR135b have one putative site in the *DEPTOR* 3′UTR (Fig. 5c). To determine whether *DEPTOR* was a direct target of those miRNAs, we carried out luciferase reporter assays. The 3′UTR of *DEPTOR* harboring the sequence complementary to the miR642a or miR135b seed sequence was cloned in a reporter plasmid vector referred to as wild-type (WT). In parallel, a 3′UTR *DEPTOR* fragment containing mutant sequences (MUT) of the seed site of the two miRNAs was cloned in the same reporter plasmid. *DEPTOR* 3′UTR WT and MUT luciferase constructs were then transfected into HEK293 cells along with miR-135b/miR-642a or negative control (NC) miRNA, and luciferase activity was determined. We found that luciferase activity of cells cotransfected with WT *DEPTOR* 3′UTR and miR135b or miR642a was significantly lower (*p* < 0.01) than that exhibited by cells transfected with NC control miRNA. Luciferase activity of MUT constructs was not affected by miR135b or miR642a overexpression (Fig. 5d).

### miR135b and miR642a regulate DEPTOR expression in MM

**Fig. 6:**
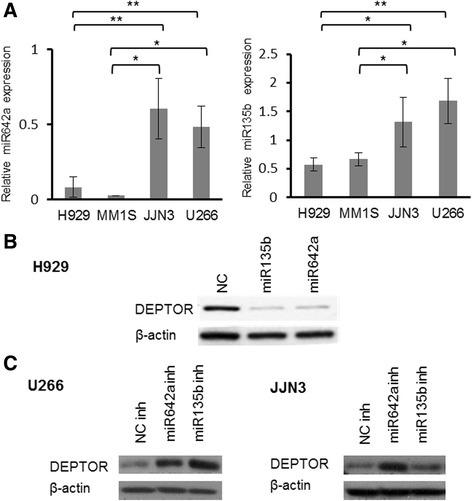
miR642a and miR135b regulate DEPTOR expression in MM. **a** miR-135b and miR-642a expression in MM cell lines measured by qRT-PCR. microRNA expression was normalized with respect to RNU43 (2^−Δct^). **b** Western blot of DEPTOR in H929 cells 72 h post-transfection with NC or miR-135b/miR-642a. **c** Western blot of DEPTOR in U266 and JJN3 transfected with NC or miR135b/miR642a inhibitors. (*p ˂ 0.05, **p ˂ 0.01, ***p ˂ 0.001)

Once *DEPTOR* had been validated as being a direct target of miR642a and miR135b, we speculated that differences in DEPTOR levels among MM cell lines could be partly related with the endogenous levels of these miRNAs. In fact, using qRT-PCR, we found that JJN3 and U266, which exhibited low levels of DEPTOR despite MAF or cyclin D1 overexpression (Fig. 6a), expressed higher levels of miR642a and miR135b than H929, which displayed high levels of DEPTOR and carried *t*(4;14) (Fig. 6a). To test this hypothesis, H929 was transfected with miR135b or miR642a and NC miRNA precursors. Clearly, lower levels of DEPTOR protein expression were found in miR135b and miR642a transfected cells compared with control cells 72 h post-transfection (Fig. 6b). To gain further evidence of the relationship between miR642a/miR135b expression and DEPTOR levels, miR642a and miR135b were specifically knocked down in U266 and JJN3 cell lines. DEPTOR protein expression was significantly higher in both cell lines after transfection with miR642a/miR135b inhibitors (Fig. 6c). Taken together, these results demonstrate that miR135b and miR642a modulate DEPTOR expression through the consensus miR135b or miR642a-binding sites in *DEPTOR* 3′UTR. These miRNAs can subsequently participate in the regulation of DEPTOR expression in MM.

### Upregulation of miR135b and miR642a results in myeloma cell dedifferentiation through the negative regulation of DEPTOR

**Fig. 7:**
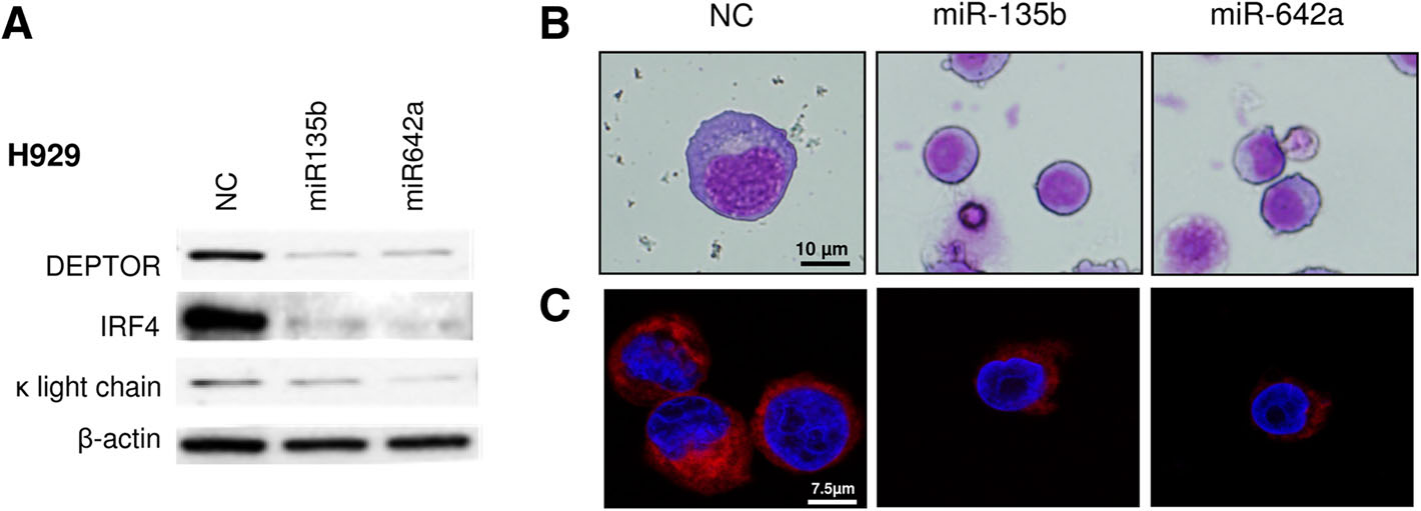
Upregulation of miR135b and miR642a results in myeloma cell dedifferentiation through negative regulation of DEPTOR. **a** Western blot of DEPTOR, IRF4, and kappa light chain in H929 cells 72 h post-transfection with NC or miR-135b/miR-642a. **b** Giemsa stain of H929 cells transfected with NC or miR-135b/miR-642a. **c** Immunofluorescence of H929 cells stained with ER tracker

Next, we were interested to determine whether downregulation of DEPTOR induced by the overexpression of miR-135b or miR-642a also led to PC dedifferentiation. Consistent with the finding after DEPTOR silencing by siRNAs, the downregulation of DEPTOR by transfection of H929 cells with miR135b or miR642a resulted in downregulation of IRF4 and k light chain proteins (Fig. 7a). In addition, DEPTOR knockdown by miR135b and miR642a overexpression led to the appearance of smaller, rounder cells with a less cytoplasm and ER content than control cells (Fig. 7b and c). These results and those obtained from siRNA experiments clearly indicated that the presence of DEPTOR is required to maintain myeloma cells at the terminal stage of differentiation.

### DEPTOR is differentially expressed in MM and its upregulation is associated with longer survival and the stage of PC maturity

**Fig. 8:**
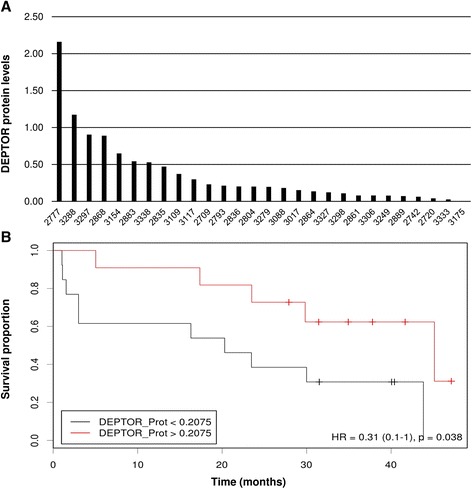
DEPTOR is differentially expressed in MM and its overexpression is associated with longer survival. **a** Levels of DEPTOR in MM patients assessed using capillary electrophoresis immunoassay. **b** Kaplan–Meier curves for progression-free survival (PFS) in 24 MM patients treated according to GEM2010

*DEPTOR* mRNA is known to be differentially expressed in MM patients [13, 28]. We confirmed these results using two different microarray studies (GSE16558, and GSE39925 at GEO repository) [27, 29]. Next, we decided to quantify DEPTOR protein levels by capillary electrophoresis immunoassay in myeloma cells from 24 MM patients treated according to the Spanish GEM2010 trial. Consistent with the mRNA expression data, we observed that levels of DEPTOR protein also differed among the myeloma samples (Fig. 8a). Interestingly, we found that the PFS was significantly longer in MM patients with high expression levels of DEPTOR than in those with low DEPTOR expression levels (*p* = 0.038) (Fig. 8b).

The differences in DEPTOR protein expression among MM patients led us to hypothesize that DEPTOR levels might be associated with the maturation state of myeloma cells of each patient, in line with our results from MMCLs. As morphological characteristics of myeloma cells are correlated with maturation stage [30], we examined the morphology of myeloma cells isolated from three patients with high DEPTOR protein levels and three with low protein levels. We observed that those cells harboring high levels of DEPTOR had an eccentric nucleus and large and extended cytoplasm, and were bigger than those exhibiting low levels of the protein (Additional file 1: Figure S3). These results support those obtained from MMCLs and indicate that a suitable level of DEPTOR is necessary for PC maturation.

## Discussion

In this study, we showed that DEPTOR, a protein overexpressed in MM [13], maintains PC differentiation. We also found that miR135b and miR642a, downregulated in the disease, modulate DEPTOR levels in MM cells.

Initial studies of PC maturation reported that the final step of PC differentiation was irreversible [9, 31–33]. However, recent studies have revealed that alterations in IRE1, XBP1, FOXP1, PAX5, or BCL6/MTA3 may reprogram PCs to previous stages of differentiation [34–37]. In keeping with these reports, we found that DEPTOR knockdown changed the transcriptional program associated with PC differentiation through the upregulation of *PAX5* and *BCL6*, which maintain the B cell program, and downregulation of IRF4, a factor that favors PC differentiation [12]. The main function of PC is the production of immunoglobulins at high rate, and for this to happen, PCs must display a highly specialized morphology with expanded cytoplasm and a more sophisticated ER network compared with B cells [38]. Our results demonstrated a clear loss of cell size and ER mass in both H929 and MM1S as a consequence of DEPTOR downregulation. Taking these results together, we propose that DEPTOR maintains the state of PCs, and its deficiency in PCs results in PC dedifferentiation. Accordingly, *DEPTOR* levels increased during the differentiation of human PCs from B cells. Peterson et al. showed that DEPTOR overexpression is necessary to maintain PI3K and AKT activation and that a reduction in DEPTOR levels leads to apoptosis [13].

Here, we found that DEPTOR inhibition, at the times and under the conditions assayed, did not affect cell survival, but reverted the commitment of PCs. A few earlier studies have found a connection between DEPTOR levels, mTORC1/mTORC2 activities, and cell differentiation. It has been reported that DEPTOR maintains stem cell pluripotency by limiting mTOR activity in undifferentiated embryonic stem cells (ESCs) [39], whereas differentiation of mouse ESCs is associated with decreased DEPTOR levels. In T cells, it has been demonstrated that mTOR drives T cell differentiation and function [40, 41]. In B cells, one study has analyzed the consequences of B cell-specific loss of the mTOR negative regulator TSC1 [42]. The authors showed that deletion of TSC1 in murine B cells and subsequent TORC1 activation led to impairment of B cell maturation. This work appears to be in agreement with our findings, in the sense that B cells would need an mTORC1 inhibitor to promote PC differentiation. However, we unexpectedly found that mTORC1/mTORC2 activities were not modified by DEPTOR silencing under our experimental conditions. The independence of mTORC1 activity was corroborated by the addition of rapamycin, an mTORC1 inhibitor that did not revert the PC dedifferentiation induced by DEPTOR knockdown. We hypothesize that DEPTOR may regulate B cell differentiation through mTOR-independent pathways. Their molecular connections with PC differentiation need to be elucidated.

DEPTOR has been found to be overexpressed in many tumor types, including breast cancer, prostate cancer, chronic myeloid leukemia, lung cancer, and MM [16]. In the latter, DEPTOR overexpression was associated with cyclin D1/D3 upregulation and especially with the presence of *MAF/MAFB* translocations [13]. The involvement of miRNAs in the pathogenesis and biology of myeloma has been suggested by several groups [27, 43, 44]. Here, we found that DEPTOR expression is also controlled by two miRNAs, miR135b and miR642a, both of which are downregulated in several MM patients [27]. Using luciferase reporter assays and gain-of-function experiments, we showed that transfection of miR135b and miR642a decreased DEPTOR levels in myeloma cells. Moreover, inhibition of miR135b and miR642a in two MMCLs exhibiting high levels of expression of both miRNAs and a low level of expression of DEPTOR, despite displaying MAF or cyclinD1 upregulation, resulted in DEPTOR overexpression. We observed that DEPTOR downregulation induced by miR135b and miR642a ectopic expression also reverted the transcriptional program of the myeloma cells and reduced cell size and ER mass, similarly to the results obtained from DEPTOR silencing by siRNA. These findings emphasize the role that these miRNAs play in regulating DEPTOR expression.

It was of particular note that DEPTOR protein levels in myeloma cells varied from patient to patient, and that its upregulation was clearly associated with longer PFS. Interestingly, high levels of DEPTOR expression have previously been associated with the prediction of response to thalidomide in MM [28]. This observation is consistent with the fact that the patients included in our study received a treatment regimen that contained lenalidomide, a thalidomide-like drug. On the other hand, we have shown that DEPTOR induces PC maturation, and it has been reported that the maturation of myeloma cells is associated with sensitivity to anti-myeloma agents [30, 34, 45–47], including lenalidomide [45]. In fact, plasma cell maturity seems to be an indicator of good prognosis in MM [47].

## Conclusions

Overall, our results show that high levels of DEPTOR result in more mature myeloma cells that would be more sensitive to therapeutic agents. They suggest the merit of further investigations to test the potential of DEPTOR levels as an indicator of maturation and as a predictive biomarker of sensitivity to anti-myeloma therapy.

## Additional file

Additional file 1: Table S1. List of oligonucleotide sequences used for 3′UTR luciferase constructs. **Figure S1.** DEPTOR knockdown did not alter cell viability. a Proliferation of myeloma cells 48 h after transfection with DEPTOR siRNA. Data are expressed as means of three independent experiments ± SD. Proliferation of cells transfected with siNT was taken as 100%, and values obtained in DEPTOR-silenced cells were normalized accordingly. b Percentage of apoptosis after DEPTOR knockdown in H929 and MM1S. Right panel shows representative dot plots. **Figure S2.** Bioinformatic identification of miRNAs that regulate DEPTOR expression. Venn diagram showing numbers of miRNAs predicted to target DEPTOR by the indicated five databases. **Figure S3.** Cell morphology and size in MM patients with different DEPTOR levels a Giemsa stain of three MM patients. b Average maximum diameter of MM cells measured from patients harboring high (*n* = 3) and low (*n* = 3) DEPTOR levels. At least 50 cells per experiment were counted. (**p* ˂ 0.05, ***p* ˂ 0.01, ****p* ˂ 0.001). (DOCX 1346 kb)

## Abbreviations

3′UTR: 3′-untraslated region
BCs: B cells
ER: Endoplasmic reticulum
miRNA: micro RNA
MM: Multiple myeloma
MMCL: Human multiple myeloma cell lines
MUT: Mutant sequence
NC: Negative control
NPCs: Normal PCs
PCs: Plasma cells
qRT-PCR: Quantitative real-time polymerase chain reaction
siRNA: Small interfering RNA
UPR: Unfolded protein response
WT: Wild-type sequence
