# Supplementary material for: DEPTOR maintains plasma cell differentiation and favorably affects prognosis in multiple myeloma
Source: J Hematol Oncol. 2017 Apr 18;10:92. doi: 10.1186/s13045-017-0461-8 (PMC5395780; doi:10.1186/s13045-017-0461-8)
Supplement: Additional file 1: Table S1. — List of oligonucleotide sequences used for 3′UTR luciferase constructs. Figure S1. DEPTOR knockdown did not alter cell viability. a Proliferation of myeloma cells 48 h after transfection with DEPTOR siRNA. Data are expressed as means of three independent experiments ± SD. Proliferation of cells transfected with siNT was taken as 100%, and values obtained in DEPTOR-silenced cells were normalized accordingly. b Percentage of apoptosis after DEPTOR knockdown in H929 and MM1S. Right panel shows representative dot plots. Figure S2. Bioinformatic identification of miRNAs that regulate DEPTOR expression. Venn diagram showing numbers of miRNAs predicted to target DEPTOR by the indicated five databases. Figure S3. Cell morphology and size in MM patients with different DEPTOR levels a Giemsa stain of three MM patients. b Average maximum diameter of MM cells measured from patients harboring high (n = 3) and low (n = 3) DEPTOR levels. At least 50 cells per experiment were counted. (*p ˂ 0.05, **p ˂ 0.01, ***p ˂ 0.001). (DOCX 1346 kb) [file 13045_2017_461_MOESM1_ESM.docx]

**Additional files:**

**Additional file 1:** Table S1. List of oligonucleotide sequences used for 3´UTR luciferase constructs.

Figure S1. DEPTOR knockdown did not alter cell viability. **a** Proliferation of myeloma cells 48 h after transfection with DEPTOR siRNA. Data are expressed as means of three independent experiments ± SD. Proliferation of cells transfected with siNT was taken as 100% and values obtained in DEPTOR-silenced cells were normalized accordingly. **b** Percentage of apoptosis after DEPTOR knockdown in H929 and MM1S. Right panel shows representative dot plots.

Figure S2. Bioinformatic identification of miRNAs that regulate DEPTOR expression. Venn diagram showing numbers of miRNAs predicted to target DEPTOR by the indicated 5 databases.

Figure S3. Cell morphology and size in MM patients with different DEPTOR levels **a** Giemsa stain of 3 MM patients. **b** Average maximum diameter of MM cells measured from patients harboring high (*n* =3) and low (*n* = 3) DEPTOR levels. At least 50 cells per experiment were counted. (* *p* ˂ 0.05, ** *p* ˂ 0.01, *** *p* ˂ 0.001).

Table S1. List of oligonucleotide sequences used for 3´UTR luciferase constructs.

| **Sequence (5´- 3´)** |
| --- |
| **DEPTOR-miR135b WT** |
| Forward AAACAAGCATTCAAGTGCTTAAAAGCCATAAAAAATGACTTCTTAATTT  Reverse ctagaaaTTAAGAAGTCATTTTTTATGGCTTTTAAGCACTTGAATGCTTGTTT |
| **DEPTOR-miR135b MUT** |
| Forward AAACAAGCATTCAAGTGCTTAAAAGCTGCGAAAAATGACTTCTTAATTT  Reverse CTAGAAATTAAGAAGTCATTTTTCGCAGCTTTTAAGCACTTGAATGCTTGTTT |
| **DEPTOR-miR642a WT** |
| Forward AAACTCCAGTGGCCTGTGGGTGAGGGAAGCCAGAATGACACAAAT  Reverse CTAGATTTGTGTCATTCTGGCTTCCCTCACCCACAGGCCACTGGAGTTT |
| **DEPTOR-miR642a MUT** |
| Forward AAACTCCAGTGGCCTGTGGGTAGAAAGGACCAGAATGACACAAAT  Reverse CTAGATTTGTGTCATTCTGGTCCTTTCTACCCACAGGCCACTGGAGTTT |


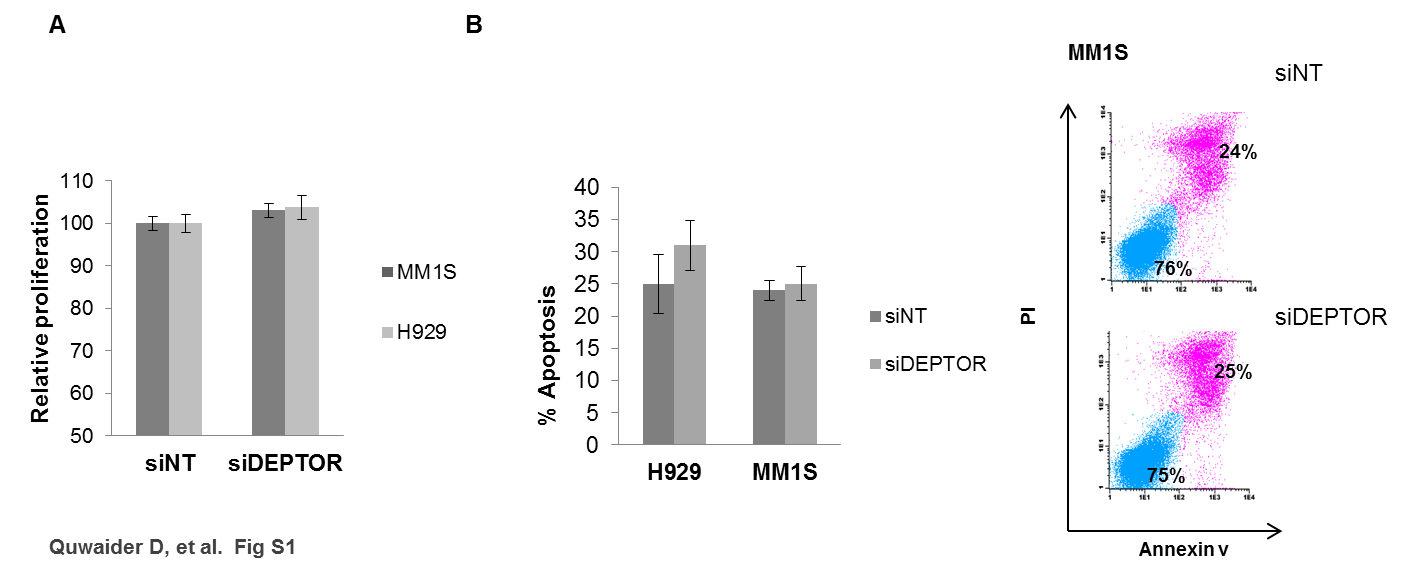


Figure S1. DEPTOR knockdown did not alter cell viability. **a** Proliferation of myeloma cells 48 h after transfection with DEPTOR siRNA. Data are expressed as means of three independent experiments ± SD. Proliferation of cells transfected with siNT was taken as 100% and values obtained in DEPTOR-silenced cells were normalized accordingly. **b** Percentage of apoptosis after DEPTOR knockdown in H929 and MM1S. Right panel shows representative dot plots.


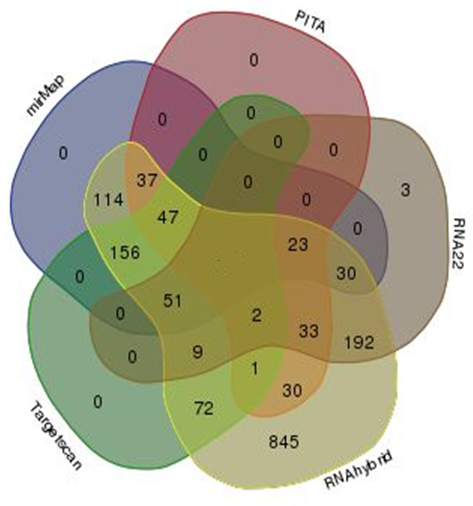


**47**

miRNA-*DEPTOR* interaction prediction algorithms

**Quwaider D, et al. Figure S2**

Figure S2. Bioinformatic identification of miRNAs that regulate DEPTOR expression. Venn diagram showing numbers of miRNAs predicted to target DEPTOR by the indicated 5 databases.


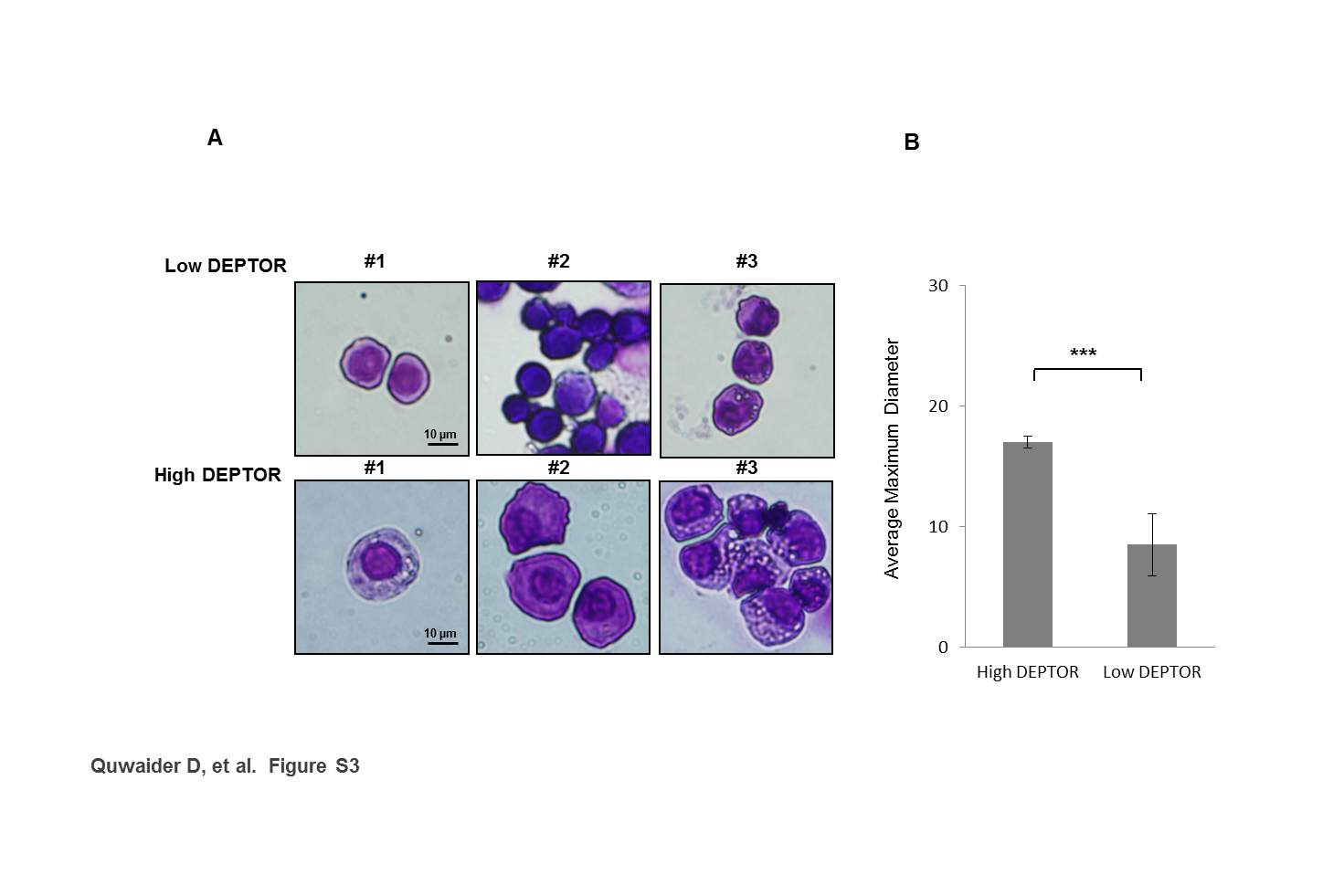


Figure S3. Cell morphology and size in MM patients with different DEPTOR levels **a** Giemsa stain of 3 MM patients. **b** Average maximum diameter of MM cells measured from patients harboring high (*n* =3) and low (*n* = 3) DEPTOR levels. At least 50 cells per experiment were counted. (* *p* ˂ 0.05, ** *p* ˂ 0.01, *** *p* ˂ 0.001).
